# Ampk regulates IgD expression but not energy stress with B cell activation

**DOI:** 10.1038/s41598-019-43985-y

**Published:** 2019-06-03

**Authors:** Lynnea R. Waters, Fasih M. Ahsan, Johanna ten Hoeve, Jason S. Hong, Diane N. H. Kim, Aspram Minasyan, Daniel Braas, Thomas G. Graeber, Thomas A. Zangle, Michael A. Teitell

**Affiliations:** 1 Molecular Biology Interdepartmental Program, UCLA, Los Angeles, CA 90095 USA; 2 Department of Pathology and Laboratory Medicine, UCLA, Los Angeles, CA 90095 USA; 3 Department of Molecular and Medical Pharmacology, UCLA, Los Angeles, CA 90095 USA; 4 UCLA Metabolomics Center, UCLA, Los Angeles, CA 90095 USA; 5 Crump Institute for Molecular Imaging, UCLA, Los Angeles, CA 90095 USA; 6 Department of Bioengineering, UCLA, Los Angeles, CA 90095 USA; 7 Jonsson Comprehensive Cancer Center, UCLA, Los Angeles, CA 90095 USA; 8 California NanoSystems Institute, UCLA, Los Angeles, CA 90095 USA; 9 Broad Stem Cell Research Center, UCLA, Los Angeles, CA 90095 USA; 10 Department of Chemical Engineering, University of Utah, Salt Lake City, UT 84112 USA; 11 Huntsman Cancer Institute, University of Utah, Salt Lake City, UT 84112 USA; 12 Department of Pediatrics, UCLA, Los Angeles, CA 90095 USA

**Keywords:** Metabolomics, Metabolomics, B cells, B cells

## Abstract

Ampk is an energy gatekeeper that responds to decreases in ATP by inhibiting energy-consuming anabolic processes and promoting energy-generating catabolic processes. Recently, we showed that Lkb1, an understudied kinase in B lymphocytes and a major upstream kinase for Ampk, had critical and unexpected roles in activating naïve B cells and in germinal center formation. Therefore, we examined whether Lkb1 activities during B cell activation depend on Ampk and report surprising Ampk activation with *in vitro* B cell stimulation in the absence of energy stress, coupled to rapid biomass accumulation. Despite Ampk activation and a controlling role for Lkb1 in B cell activation, Ampk knockout did not significantly affect B cell activation, differentiation, nutrient dynamics, gene expression, or humoral immune responses. Instead, Ampk loss specifically repressed the transcriptional expression of *IgD* and its regulator, *Zfp318*. Results also reveal that early activation of Ampk by phenformin treatment impairs germinal center formation but does not significantly alter antibody responses. Combined, the data show an unexpectedly specific role for Ampk in the regulation of IgD expression during B cell activation.

## Introduction

B lymphocyte activation is an early step in a humoral immune response, whereby a naïve B cell with a unique antigen receptor recognizes its cognate antigen to trigger growth, division, and differentiation. Following activation, selected B cells can develop into long-lived plasma cells that secrete antigen-specific antibodies to fight infections^1^. During receptor-mediated activation, B cells undergo class switch recombination (CSR), also called immunoglobulin isotype switching, to modify the type of B cell antigen receptor (BCR) expressed by the B cell, such as IgM or IgG1 isotypes^2^. Many of the early signaling events linked to engagement of the BCR are well studied^3^. Recently, we reported a role for the tumor suppressor Lkb1 in B cell activation: *Lkb1* knockout (KO) caused spontaneous B cell activation *in vivo* without specific added antigenic stimulation, resulting in a robust T cell-dependent germinal center (GC) reaction^4,5^. This result was interesting because Lkb1 signaling had not been previously implicated in B cell activation and few models of spontaneous GC formation exist^6^. We therefore sought to determine the mechanism(s) whereby Lkb1 controls B cell activation.

Lkb1 phosphorylates 14 different related kinase family member proteins to control many cellular functions including protein synthesis and cell growth, cell polarity, and metabolism^7^. We elected to examine one of these 14 major downstream Lkb1 targets, 5′ AMP-activated protein kinase (Ampk). Ampk is an energy sensor that couples metabolism with nutrient availability during periods of energetic stress, as might occur during rapid B cell expansion and differentiation^8^. Ampk does this by sensing increasing levels of ADP or AMP with reducing levels of ATP in a cell, which triggers the phosphorylation of well characterized substrate proteins including Tsc2, Acc1/2, and Tbc1d1 to inhibit protein synthesis, promote fatty acid oxidation, upregulate glycolysis, and restore overall cell energy balance^9^. While Lkb1 is the major upstream kinase for Ampk, other upstream kinases also phosphorylate Ampk including CamKK2 and Tak1^10–12^. In T cells, CD3 ligation results in rapid Ampk activation in a calcium- and CamKK2-dependent manner^13^, and Ampk activation declines in proliferating normal T cells^14^; however, the Ampk activation pattern in B cells is unknown.

Studies of Lkb1 and Ampk have shown overlapping but also unique functions in hematopoiesis. For example, Lkb1 maintains hematopoietic stem cell quiescence by regulating metabolism and the cell cycle using Ampk-dependent and -independent mechanisms^15–17^. In T cells and thymocytes, Lkb1 deletion reduced peripheral T cells and decreased T cell proliferation when stimulated *in vitro*. However, while loss of Ampk in T cells also led to metabolic and activation defects, it did not fully recapitulate the loss of Lkb1^18–20^. There is only one reported study of Ampk in B cells, which showed that a whole mouse knockout of Ampk left isolated B and T cells unable to survive *in vitro* under oxidative stress when exposed to the ATP synthase inhibitor, oligomycin^21^. Given the unexpected role for Lkb1 loss in B cells in triggering a GC reaction, we sought to determine role(s) for Ampk during B cell activation.

## Results

### Ampk activation during B cell stimulation

**Figure 1:**
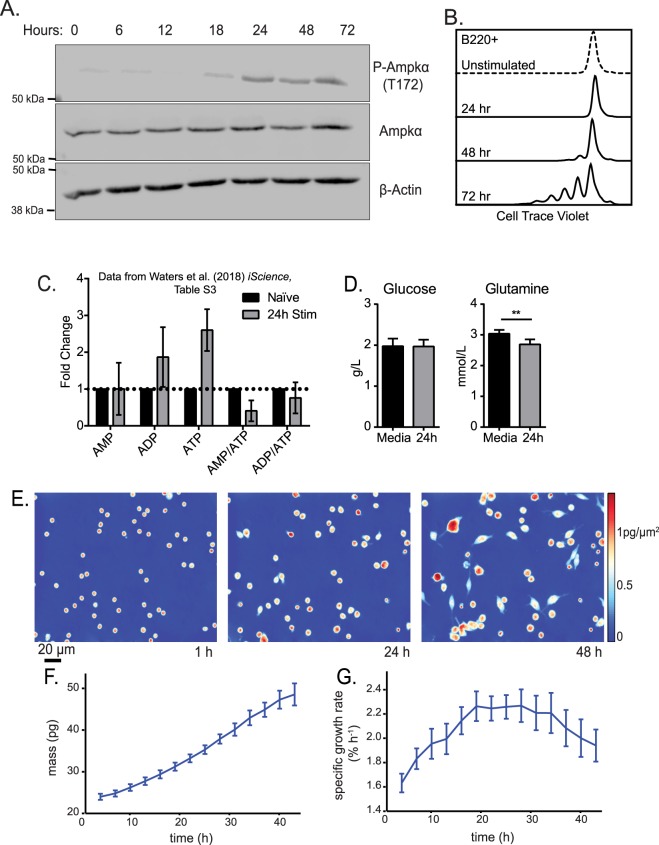
Activation of Ampk upon stimulation of B cells is independent of energy stress and does not result in lowered biomass accumulation. (**A**) Representative time course western blot for phosphorylated Ampkα (T172), Ampkα, and β-tubulin during anti-CD40 plus IL-4 stimulation of B cells. Image was cropped for clarity, full-length blots/gels are presented in Supplementary Fig. 1. (**B**) Representative flow cytometry of B220+ B cells at 0, 24, 48 and 72 hours post anti-CD40 plus IL-4 stimulation stained with Cell Trace Violet. (**C**) Relative fold change in previously published UHPLC- MS metabolomics dataset^24^ for adenine nucleotides from 24 hours post stimulation with anti-CD40 plus IL-4 relative to naïve B cells (*n* = 3). (**D**) Measurement of extracellular glucose and glutamine at 24 hours post stimulation with anti-CD40 plus IL-4 (*n* = 3). (**E**) Live cell interferometry (LCI) images of anti-CD40 plus IL-4 activated B cells 1, 24, and 48 hours post stimulation showing significant growth, as confirmed by (**F**) a significant increase in average cell mass over time, binned into 3 h increments. (**G**) Average specific growth rate, computed as the instantaneous slope of mass over time for each cell normalized by cell mass, shows a peak at 24 hours, coincident with Ampk inactivation (*n* = 5 independent experiments of at least 200 cells). Data represent mean ± SD (**C**,**D**) or SEM (**F**,**G**). *P* values determined by 2-way ANOVA with Bonferroni correction for multiple comparisons (**C**) or an unpaired two-tailed Student’s *t*-test (**D**). **P ≤ 0.01.

Initially, we investigated whether Ampk, a major downstream target of Lkb1, was required for B cell activation^4,5^. Previous studies in T cells showed Ampk activation after T cell receptor stimulation^13^. We examined the phosphorylation of Ampk at T172, a marker residue for Ampk activation^22^ and determined that Ampk activation occurs between 18–24 hours post-stimulation of B cells with anti-CD40 antibody plus interleukin (IL)-4 that persists at least through 72 hours (Fig. 1A). Activation of Ampk should initiate cellular processes that halt the accumulation of biomass required for cell division^9^. Instead, anti-CD40 plus IL-4 stimulated B cells to divide rapidly between 48–72 hours (Fig. 1B). Ampk activation with energy stress has been reported many times and occurs by sensing decreasing amounts of ATP linked to increasing ratios of AMP:ATP and ADP:ATP^23^. Therefore, we examined a previously published dataset of nucleotide metabolite levels at 24 hours post-stimulation. UHPLC-MS metabolomics data of ^13^C_6_-glucose nutrient labeling during initial B cell activation showed unexpected AMP:ATP and ADP:ATP ratios declining at 24 hours with ATP steady-state levels significantly increasing (Fig. 1C)^24^. Additional measurements of extracellular nutrients shows maintenance of high levels of both glucose and glutamine in the culture medium (Fig. 1D), indicating that Ampk activation occurs in stimulated B cells during energy replete conditions.

Since Ampk activation typically inhibits protein, lipid, and additional biosynthetic processes, we turned to live cell interferometry, a form of quantitative phase microscopy, as a high precision and reproducible approach to quantify changes in biomass accumulation during early B cell activation^25^ (Fig. 1E). We discovered that isolated naïve mouse B cells steadily increase cell biomass over the first 48 hours of anti-CD40 plus IL-4 stimulated activation (Fig. 1F). Interestingly, the specific growth rate calculated as a percentage of biomass change per hour accelerates for the first 18 hours and then plateaus (Fig. 1G). This growth rate peak coincides with the timing of Ampk activation (Fig. 1A), although despite Ampk activity B cells continue to acquire biomass at the peak (plateau) rate. This growth rate profile suggests that Ampk activation may damp further growth rate acceleration, perhaps as a rate-limiting step, but does not impede the overall growth of B cells.

### Ampk is not required for B cell activation or differentiation

**Figure 2:**
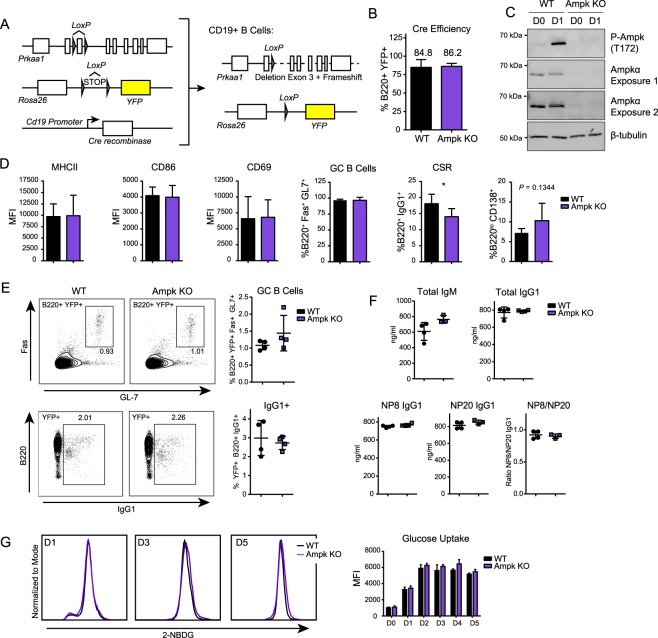
Ampk deletion does not significantly impair B cell function. (**A**) Strategy for generating B-cell lineage specific knockout of *Prkaa1* by crossing *CD19-Cre* recombinase (JAX: 006785) mice with *Prkaa1*^*fl/fl*^ (JAX: 014141) mice and *Rosa26 lox-STOP-lox YFP* (JAX: 006148) mouse lines, yielding mice where CD19+ B cells lack the catalytic Ampkα subunit. (**B**) Quantification of Cre-recombinase efficiency by measurement of percentage of B220+ B cells that have YFP expression by flow cytometry in WT and Ampk KO B cells (*n* = 3). (**C**) Representative Western blot for P-Ampkα (T172), Ampkα and β-tubulin in WT and Ampk KO mice. Two exposures are shown for Ampkα to illustrate some remaining Ampk expression. Image was cropped for clarity, full-length blots/gels are presented in Supplementary Fig. 2. (**D**) Flow cytometry of WT and Ampk KO B cells during activation with anti-CD40L plus IL-4, including activation markers (MHCII, CD86, CD69) at 24 hours, GC differentiation (GC, %B220+ Fas+ GL7+) and CSR (%B220+ IgG1+) at day 3, and plasmablast differentiation (%B220^lo^ CD138+) at day 5 (*n* = 5 for MHCII, CD86, CD69, and PB, *n* = 6 for GC, and *n* = 7 for CSR). (**E**) Flow cytometry of total splenocytes after immunization. Representative plots and quantification of GC differentiation (GC B Cells, B220^+^ Fas^+^ GL7^+^) and CSR (B220^+^ IgG1^+^) 14 days post-immunization with NP-(28)-CGG (*n* = 4). (**F**) Total IgM, total IgG1, NP8 IgG1, NP20 IgG1, and NP8/NP20 IgG1 ratio in serum 14 days after immunization of WT and Ampk KO mice with NP-(28)-CGG (*n* = 4). (**G**) Representative flow cytometry for 2NBDG glucose uptake at day 1, 3, and 5 post stimulation with anti-CD40 plus IL-4, and quantification at day 0 through 5 in WT and Ampk KO B cells (*n* = 3). Data represent mean ± SD (**B**,**D**–**G**). *P* values determined by Student’s *t*-test (**B**,**D**–**G**), *P ≤ 0.05.

It was a surprise that Ampk was activated in stimulated naïve mouse B cells because a major role for Ampk in inhibiting lipid and protein synthesis seems incompatible with the high growth rate and cell division of activated B cells. Thus, we sought to determine whether Ampk was required for B cell activation and differentiation by generating a mouse model with B cell specific deletion of Ampk. Because Ampkα1, encoded by *Prkaa1*, is the only α subunit expressed in trimeric Ampk proteins in mature B cells^26^, deletion of this catalytic subunit abolishes all Ampk activity. We generated a B cell specific Ampkα1 KO mouse line by crossing *Prkaa1*^fl/fl^ mice with *CD19-Cre* recombinase driver mice to delete Ampk activity in post-pro/pre B cells. To monitor deletion efficiency, we crossed mice with a *Rosa26 lox-STOP-lox YFP* reporter allele (Fig. 2A). Deletion efficiency measured by YFP+ B220+ B cells was >80% in both WT (*Prkaa1*^+*/*+^ × *CD19-Cre*) and Ampk KO (*Prkaa*^*fl/fl*^ × *CD19-Cre*) mice (Fig. 2B), suggesting that there is no B cell survival disadvantage with loss of Ampk, in contrast to a major B cell survival disadvantage with Lkb1 loss^4^. Analysis of lysates from naïve and anti-CD40 plus IL-4 stimulated WT and Ampk KO B cells confirmed loss of Ampkα1 protein and abolishment of Ampk activity in Ampk KO B cells (Fig. 2C). Use of a pan-Ampk alpha (α1 and α2) antibody also allows us to conclude that there was no compensatory expression of the Ampkα2 subunit upon deletion of Ampkα1.

To determine whether Ampk deletion affects B cell activation, we stimulated isolated WT and Ampk KO B cells with anti-CD40 plus IL-4 and assessed activation and differentiation of YFP+ B cells. Surprisingly, despite Ampk activation at 24 hours, no changes in the levels of expression of activation markers CD69, CD86 or MHCII occur for the Ampk KO B cells compared to WT B cells. At later time points, no changes in GC-like B cell differentiation at day 3 and a minor reduction in CSR to IgG1 occurred in Ampk KO compared to WT B cells. By day 5, there was a small trending increase in plasmablast differentiation in Ampk KO versus WT B cells (Fig. 2D). These data strongly contrast with deletion of Lkb1 in B cells, which causes opposing results including increased activation marker expression and GC-like B cell differentiation, an increase in CSR, and a decrease in plasmablast differentiation^4^. Together, the results suggest that Ampk is not required for normal B cell function and is not required for Lkb1-dependent B cell phenotypes.

### Ampk does not control a humoral immune response *in vivo*

We next examined whether Ampk deletion from B cells affects antibody responses *in vivo*. A prior study showed that whole mouse KO of Ampk did not affect IgG responses to Ars-KLH antigen *in vivo*^21^; however, other potential effects on the GC reaction, CSR, or antibody specificity were not examined. We inoculated Ampk KO and WT mice with the T cell-dependent antigen, NP-(28)-CGG, and analyzed B cells on day 14 post-immunization. We observed no differences in GC formation or CSR (Fig. 2E), and serum IgG1 levels were similar between WT and Ampk KO mice, with a slight but statistically insignificant difference in total IgM (Fig. 2F). Multivalent NP antigen enables detection of highly specific antibodies by probing for binding to specific NP molar ratios, with fewer NP molecules revealing higher specificity responses. Our data show that both broad spectrum and highly specific IgG1 against NP antigen were similar in WT and Ampk KO mice (Fig. 2F). These results show that Ampk is dispensable for a T cell-dependent humoral immune response, although Ampk may be critical in other contexts, such as T cell-independent, mucosal, or antiviral immune responses.

### Ampk is dispensable for activation-induced glucose uptake

While we did not detect differences in differentiation patterns of WT and Ampk KO B cells, B cell activation in response to a range of triggering stimuli causes increased glucose import^24,27,28^ and the deletion of Ampk in T effector cells reduces glucose uptake *in vivo*^29^. Therefore, we hypothesized that Ampk KO B cells might have defects in glucose uptake. To test this postulate, we utilized a fluorescent glucose analog, 2-NBDG, to measure glucose import in B cells from WT and Ampk KO mice that lack the *Rosa26 lox-STOP-lox* YFP tracer, because YFP and 2-NBDG fluorophore emission spectra overlap (527 nm and 540 nm, respectively). Unexpectedly, there was no difference between Ampk KO and WT B cells in glucose uptake during 5 days of B cell activation (Fig. 2G). Thus, Ampk activation does not regulate glucose import into activated B cells, suggesting an alternative glucose import mechanism.

### Ampk does not affect glucose or glutamine B cell nutrients

**Figure 3:**
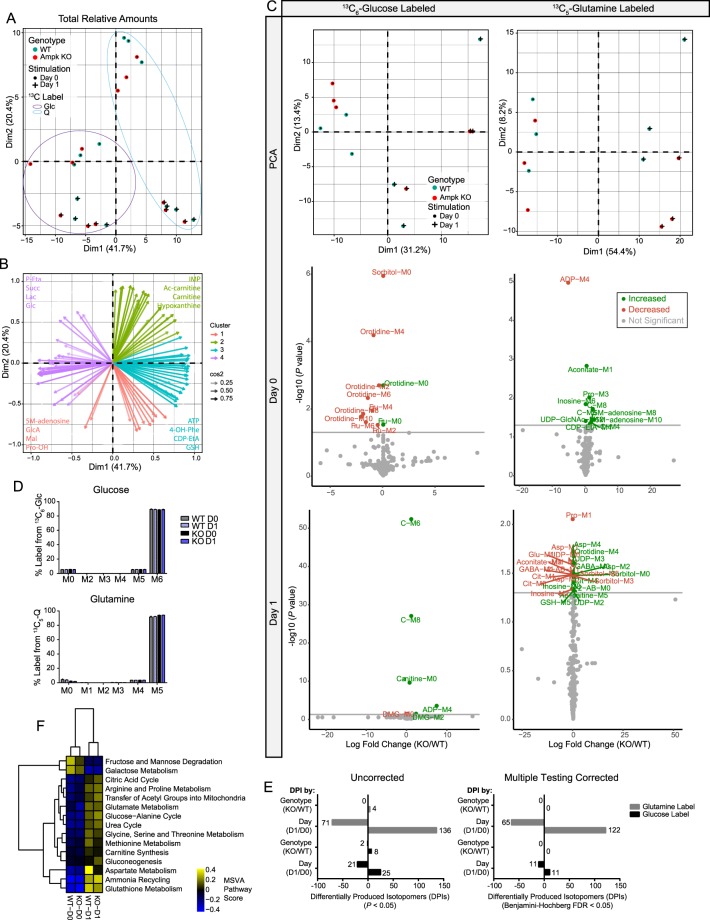
Ampk does not control metabolism during B cell stimulation. (**A**) PCA from UHPLC-MS metabolomics analysis of total metabolites isolated from naïve and stimulated B cells from WT and Ampk KO mice. (**B**) K-means clustering analysis of variable loading plots, indicating the contribution of metabolites to variation across principal components in four groups (k = 4). Projection arrow tips indicate Pearson correlation of the listed metabolite. Transparency denotes cosine^2^ value or the strength of metabolite representation. Shown are the top 4 metabolites contributing to each cluster. (**C**) Principal component analysis and volcano plots of UHPLC-MS metabolomics of ^13^C_6_-glucose labeled or ^13^C_5_-glutamine labeled isotopomers (MID) in naïve and stimulated B cells from WT and Ampk KO mice. Plotted are non-corrected *P* values for KO/WT amounts of specific isotopomers (*n* = 3). (**D**) Isotopomer distribution of labeled glucose or labeled glutamine in naïve or stimulated WT and Ampk KO B cells shows uptake of labeled respective carbon source. (**E**) Quantification of Differentially Produced Isotopomers (DPIs) analyzed both by day and by genotype shows no DPIs between genotypes, only between time points for uncorrected or Benjamini-Hochberg corrected *P* values < 0.05. (**F**) Heatmap of significant (Benjamini-Hochberg FDR adjusted *P* value < 0.05) metabolite set variation analysis (MSVA) pathway activity scores across WT and Ampk KO naïve and stimulated B cells for curated KEGG metabolic pathways. Data represent the mean ± SD (**D**) of relative metabolite amounts from *n* = 3 independent experiments. Data from separate glucose and glutamine labeling experiments were pooled, and relative amounts analyzed for (**A**,**B**). *P* values determined by Student’s t test (**C**), 2-way ANOVA with Bonferroni correction for multiple comparisons (**D**), or empirical Bayes with Benjamini-Hochberg correction for multiple comparisons (**F**).

**Figure 4:**
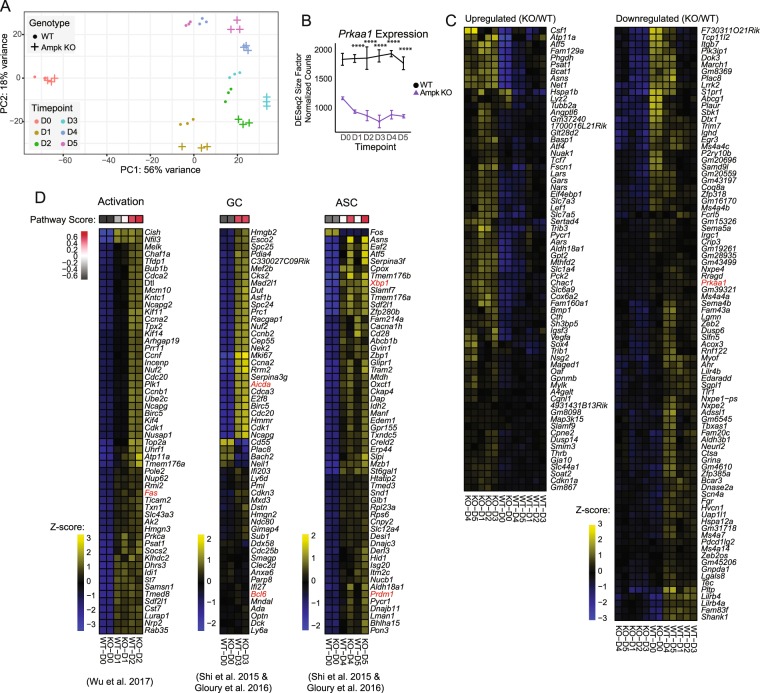
B cell activation and differentiation expression signatures are maintained despite loss of Ampk. (**A**) PCA from bulk RNA sequencing analysis of total RNA isolated from day 0 through day 5 stimulated B cells from WT and Ampk KO mice. PC1 and PC2 are shown. (**B**) Kinetic time course expression plot of *Prkaa1* during stimulation. (**C**) Heat map of KO signature gene expression across averaged samples. KO signature was generated as the intersection of all KO/WT DEGs at each time point from day 1 to day 5 of stimulation (adjusted *P* value < 0.05, abs(log_2_FC) >0.5 at each time point comparison). (**D**) Heat maps for B cell activation, germinal center, and ASC gene signatures from^32,33,55^. Genes shown were selected as the top 55 differentially expressed from the Shi 2015 signatures between WT day 0 and day 2 (activation), WT day 0 and day 3 (GC), and WT day 0 and day 5 (ASC), with *Prdm1* included *post hoc*. Pathway scores represent total GSVA enrichment of the respective Shi 2015 signatures for each comparison (adjusted *P* value < 0.05). Heat map values represent row z-score (*n* = 3 each WT and KO for each time point). Adjusted *P* values determined by Wald test (**B**). ****P ≤ 0.0001.

While we did not detect differences in glucose uptake in Ampk KO B cells, Ampk also regulates other metabolic pathways to regulate energy stress, and Ampk activation at 24 hours of stimulation may impact nutrient choice or routing without affecting B cell differentiation or antibody responses. Therefore, we performed metabolomics profiling with ^13^C_6_-glucose and ^13^C_5_-glutamine in resting and anti-CD40 plus IL-4 stimulated WT and Ampk KO B cells (Table S1). Principal component analysis (PCA) of total intracellular metabolites revealed that the major segmentation was from differences between stimulation time points, and not from differences between genotypes within each time point (Fig. 3A). We generated correlation circle plots by fitting each of the metabolites to 4 vectors based on the observed clustering (Fig. 3B), which indicated metabolites that are known to be changed upon activation, including increases in ATP with activation and decreases in the AMP/ATP ratio, consistent with prior results (Fig. 1C)^24^. With no significant differences in total metabolites between WT and Ampk KO B cells at rest or for any stimulated time point, we next analyzed specific ^13^C-isotopomer labeling. PCA of molecular IDs for isotopomers (Fig. 3C, top) derived from the glucose label (left) or glutamine label (right) showed similar segmentation to the total metabolites, with the largest differences from stimulation time point rather than from genotype. We then assessed whether any biologically relevant metabolites were different between WT and Ampk KO at each time point. As a discovery tool, we plotted the non-corrected *P*-values for naïve, resting (middle row) and anti-CD40 plus IL-4 stimulated (bottom row) B cells labeled with glucose (left) or glutamine (right) (Fig. 3C). The data failed to identify any biologically relevant differentially produced isotopomers (DPIs) linked to known functions of Ampk. These labeling patterns occurred despite >90% uptake of labeled glucose or glutamine in each respective experiment for all conditions examined (Fig. 3D). To further investigate whether there were any DPIs between WT and Ampk KO B cells irrespective of time point, we calculated *P*-values and could not identify metabolites below the corrected false discovery rate of 0.05 (Fig. 3E). We considered that some broad metabolic differences may not be discernible at the individual DPI level, and small changes may accumulate and reveal deficiencies or surpluses in whole pathways. We therefore performed Metabolite Set Variation Analysis (MSVA) utilizing curated KEGG metabolic pathways and again identified significant changes occurred only between naïve and stimulated B cells, with no separation by genotype (Fig. 4F). Together, these results show that Ampk is not modulating metabolism during B cell activation, despite known metabolic roles related to glucose metabolism.

### Ampk regulates IgD levels but not transcripts controlling B cell fate

Additional canonical Ampk substrates include Hdac4 and Hdac5, proteins that deacetylate histones and thereby regulate gene expression^30^. To assess potential differences in steady-state RNA expression, and to investigate potential alternative processes regulated by Ampk, we performed RNA-Seq in WT and Ampk KO B cells on culture days 0 through 5 using anti-CD40 plus IL-4 stimulation (Table S2). At day 0, there are no PCA plotted statistical differences between WT and Ampk KO naïve B cells. However, as B cells differentiate from day 1 through day 5, Ampk KO B cells cluster separately from their WT counterparts, but follow a similar trajectory towards differentiation (Fig. 4A). We confirmed reduced *Prkaa1* expression in Ampk KO B cells, as expected (Fig. 4B). To determine whether loss of Ampk conveyed a specific gene signature during 5 days of differentiation, we analyzed differentially expressed genes (DEGs) for days 0 through 5 of stimulation and identified 168 genes that were either consistently increased or decreased each day in Ampk KO compared to WT B cells (Fig. 4C). Pathway enrichment analysis of these 168 genes did not reveal any pathways related to B cell activation or differentiation, with the exception of 5 genes linked to apoptotic signaling in response to endoplasmic reticulum stress (*Grina*, *Atf4*, *Trib3*, *Lrrk2*, *Chac1*) (Table S2). Endoplasmic reticulum stress has an important role in plasma cell differentiation and antibody secretion^31^. Therefore, we analyzed activation, GC B cell, and antibody-secreting cell (ASC) gene expression signatures^32,33^ as previously reported in our data set (Fig. 2D). Similar to our earlier data, Ampk KO and WT B cells activate similarly and increase GC-signature transcripts equally. Expression of ASC signature genes were similar between WT and Ampk KO samples (Fig. 4D), although Ampk KO B cells showed increases in some ASC transcripts, including *Prdm1* at days 4 and 5 (Fig. 4D). This finding is consistent with a trend towards an increase in plasmablasts at day 5 (Fig. 2D), and a divergence in the PCA plot of days 4 and 5 WT versus Ampk KO B cells (Fig. 4A).

**Figure 5:**
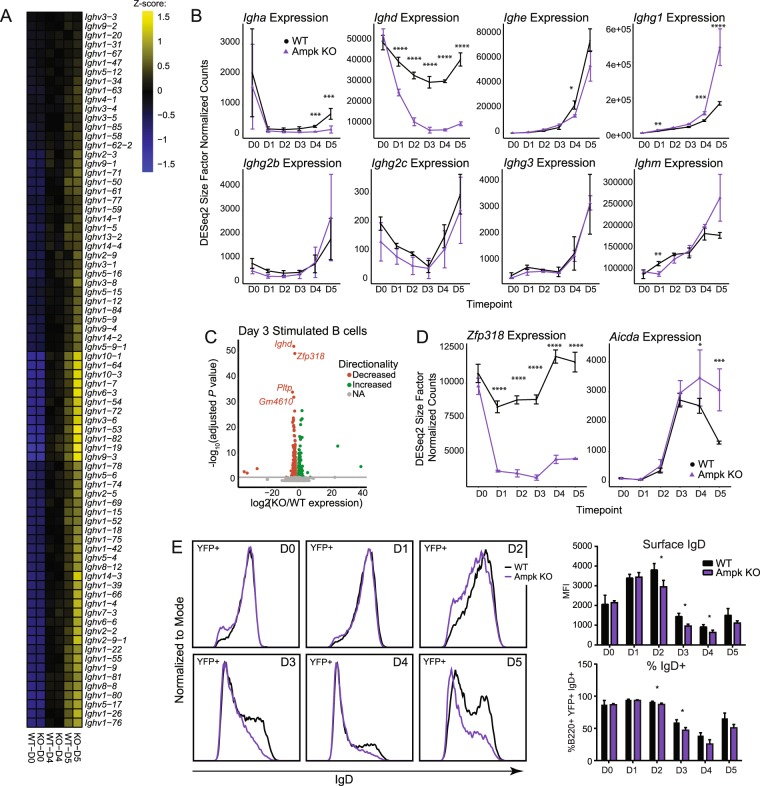
Ampk specifically regulates expression of IgD via downregulation of Zfp318 during activation. (**A**) Heat maps for immunoglobulin heavy-chain variable region expression (*Ighv*) across naïve and day 4/day 5 stimulated B cells. Genes were selected based on significant differential expression between WT naïve and day 5 stimulation (adjusted *P* value < 0.05). Values represent row z-score. (**B**) Kinetic time course expression plots for immunoglobulin heavy-chain constant region (*Igh*) expression across genotype and stimulation. (**C**) Volcano plot of differentially expressed genes between Ampk KO and WT at day 3 of stimulation. Adjusted *P* value cutoff represents values < 0.05 calculated using the Wald test following DESeq2 normalization. (**D**) Kinetic time course expression plot of *Zfp318* and *Aicda* across genotype and stimulation. (**E**) Representative flow cytometry plot of IgD at day 0 through day 5 of stimulation in WT and Ampk KO B cells and quantification by MFI (top) and % IgD (bottom) (*n* = 3 each WT and Ampk KO). *P* values were determined by Student’s t-test (**E**), adjusted *P* values were determined by Wald test (**B**,**D**) *P ≤ 0.05, **P ≤ 0.01, **P ≤ 0.001, ***P ≤ 0.0001.

Because of the potential for increased ASCs in Ampk KO mice, despite similar amounts of secreted IgM and IgG1 from *in vivo* immunizations (Fig. 2F), we evaluated the levels of immunoglobulin transcripts in WT and Ampk KO B cells. We determined that heavy chain variable (*Ighv*) region expression levels were similar for WT and Ampk KO B cells, although there was an increase in some transcripts including *Ighv6-3* and *Ighv14-3* in Ampk KO B cells (Fig. 5A). From *in vivo* immunization data, we also detected a slight but insignificant increase in secreted IgM, so we analyzed transcripts for immunoglobulin constant regions to identify potential isotype biases. Whereas *Ighm* transcripts matched our observations *in vivo* with a minor but insignificant increase at day 5 for Ampk KO versus WT B cells, we were surprised to discover that *Ighd* expression was severely repressed in Ampk KO B cells from day 1 stimulation onwards (corrected *P*-value = 9.5 × 10^−30^ at day 1) (Fig. 5B). IgD is normally co-expressed with IgM on the surface of all mature, naïve B cells, and is a relatively understudied antibody class that has potential implications in mucosal immune responses^34^. The expression of IgD is not impacted by activation-induced cytidine deaminase (AID) like most isotype switched immunoglobulins but is instead regulated by Zfp318^35,36^. Interestingly, the most robust DEGs at day 3 are *Ighd* and *Zfp318*, each of which are greater than an order of magnitude more repressed than the next most differentially expressed genes (Fig. 5C). In fact, *Zfp318* expression is repressed throughout the stimulated time course in Ampk KO B cells (Fig. 5D), coinciding with the drop in *Ighd* expression (Fig. 5B). Further supporting the specificity of Ampk loss on Zfp318 regulation of *Ighd* levels, expression of *Aicda*, encoding AID which regulates CSR, is the same in WT and Ampk KO B cells from days 0–4 and increased on day 5 of B cell activation (Fig. 5D). To confirm that the loss of *Ighd* transcripts affects IgD protein levels, we analyzed surface expression over 5 days of differentiation by flow cytometry, which shows that Ampk KO B cells have decreased IgD expression from days 2 through 5, particularly evident for the loss of IgD-high B cells (Fig. 5E). Given the limited number of DEGs, it appears that Ampk exerts highly specific control of IgD during B cell activation, likely through regulation of *Zfp318* expression.

### Pharmacological activation of Ampk

**Figure 6:**
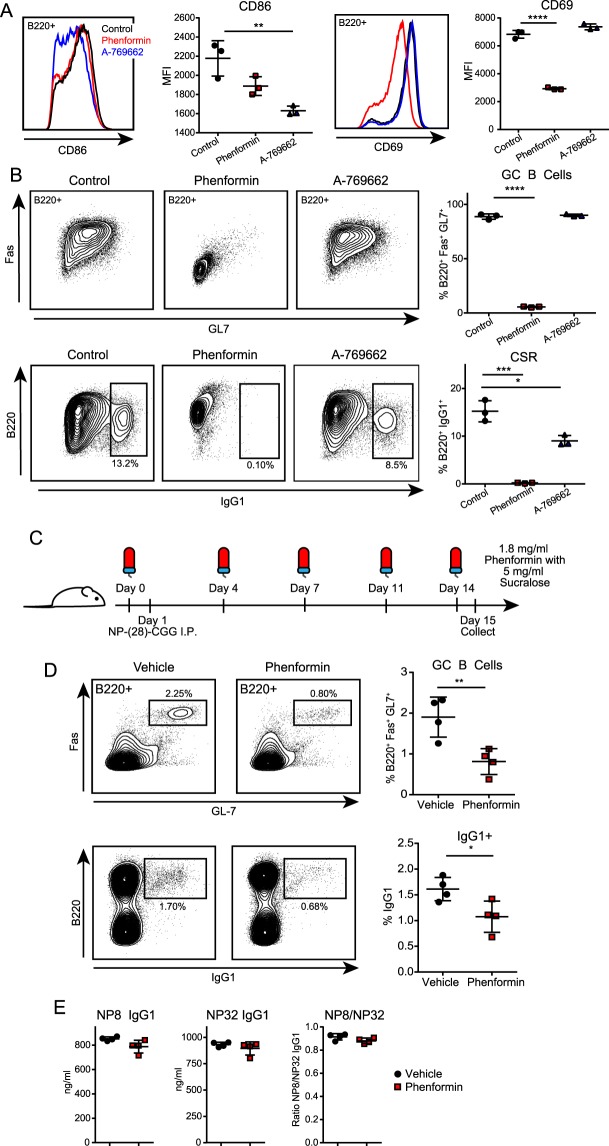
Early pharmacological activation of Ampk modifies B cell function both *in vitro* and *in vivo*. (**A**,**B**) Representative flow cytometry plots and quantification of activation markers (CD86, CD69) at 24 hours (**A**), and germinal center differentiation (GC B Cells, %B220^+^ Fas^+^ GL7^+^) and class switch recombination (CSR, %B220^+^ IgG1^+^) at day 3 (**B**) of cells during *in vitro* activation with anti-CD40 plus IL-4 and phenformin (100 µM) or A-769662 (50 µM) (*n* = 3). (**C**) Strategy for *in vivo* assessment of B cells responses with Ampk activation. Prior to immunization, mice were given phenformin or vehicle (sucralose) in their water. Water was changed 2×/week, and samples collected 14 days post immunization with NP-(28)-CGG. (**D**) Flow cytometry of total splenocytes after immunization. Representative plots and quantification of GC differentiation (GC B Cells, B220^+^ Fas^+^ GL7^+^) and CSR (B220^+^ IgG1^+^) 14 days post-immunization with NP-(28)-CGG (*n* = 4). (**E**) Anti-NP8 and anti-NP32 IgG1 serum response and NP8/NP32 IgG1 ratio by ELISA 14 days after immunization with NP-(28)-CGG (*n* = 4). Data represent mean ± SD. *P* values determined by 2-way ANOVA with Bonferroni correction for multiple comparisons (**A**,**B**), or Student’s *t*-test (**D**,**E**), *P ≤ 0.05, **P ≤ 0.01, ***P ≤ 0.001, ****P ≤ 0.0001.

The limited scope of impact for Ampk loss on B cell physiology seems surprising, so we examined whether the timing of Ampk activation could regulate B cell functions. We utilized two pharmacological activators of Ampk, phenformin and A-769662, to alter the timing of Ampk activation. Phenformin is a mitochondrial electron transport chain complex I inhibitor that activates Ampk by inhibiting ATP production, thereby increasing AMP/ATP and ADP/ATP ratios, and is an analogue of the diabetes drug metformin, whereas A-769662 is a direct and specific activator of Ampk^23^. We examined early activation of Ampk by treating B cells with each activator at the time of anti-CD40 plus IL-4 stimulation in culture and observed that both drugs decreased CD86 activation biomarker expression, but only phenformin reduced CD69 expression (Fig. 6A). Phenformin had a drastic effect on B cell differentiation by day 3 with greatly decreased CSR to IgG1 and inhibited differentiation into GC-like B cells (Fig. 6B). By contrast, A-769662 had little effect on GC-like B cell differentiation and only a slight defect in CSR (Fig. 6B). These results show that electron transport chain activity and ATP production *per se*, and not accelerated Ampk activation, are critical for B cell activation, differentiation, and CSR, in agreement with an effect mainly targeting markedly reduced *Zfp318* and *Ighd* expression levels in stimulated Ampk KO B cells (Fig. 5).

Because of recent interest in using metformin and phenformin clinically to treat B cell malignancies^37^, we further evaluated the impact of phenformin on B cell function. To determine whether the effects of phenformin *in vitro* replicate *in vivo*, we immunized mice with NP-(28)-CGG to induce a T cell-dependent humoral immune response while delivering phenformin or sucralose vehicle in the drinking water (Fig. 6C). *In vivo* results show that 14-day treatment with phenformin substantially decreased the percentage of GC B cells in the spleen and reduced the percentage of IgG1+ isotype switched B cells (Fig. 6D). Interestingly, however, mice on phenformin had similar amounts of total IgM and IgG1 in their serum (Fig. 6E). There was a minor but non-significant defect in the generation of NP8-specific high affinity IgG1 antibody in phenformin treated mice, but no effect on broader NP20 IgG1 antibodies (Fig. 6F). Overall, these findings suggest that phenformin reduces GC formation, but still allows for generation of antigen-specific antibody production.

## Discussion

Our recent results showing that loss of Lkb1 kinase signaling triggers the B cell GC reaction^4,5^ prompted studies of Ampk as a main Lkb1 target kinase during B cell activation. We found Ampk activation 24 hours after stimulation of naïve B cells with anti-CD40 antibody plus IL-4 (Fig. 1A). A key role for activated Ampk in mammalian cells is to block anabolic processes that consume energy by target protein phosphorylation in response to energy stress. To our surprise, B cell activation with rapid biomass accumulation and cell proliferation coincides with sustained Ampk activation in the absence of energy stress (Fig. 1A,B). We anticipated the opposite result, that Ampk activity would prevent biomass accumulation. While unexpected given the canonical targets and activities of Ampk, including inhibition of major anabolic targets Acc1, Tsc2, and Raptor, these findings also make sense in the biological context of B cell activation. For example, the Ampk target Raptor is essential for *Bcl6* expression and recruitment of activated B cells into GCs^38^, and inhibition of Raptor by Ampk at this time would prevent GC formation. Similarly, Ampk-driven inhibition of protein and lipid synthesis through Acc1 and Tsc2 would antagonize the need for biomass accumulation while B cells prepare for rapid division in the GC^24,39,40^. While activation of Ampk during B cell activation was unexpected due to the role of Ampk in biomass accumulation, there are precedents for Ampk activation in other immune contexts. For example, in T cells, Ampk is transiently and immediately activated after CD3 or calcium stimulation^13^, and Ampk phosphorylation declines in proliferating T effector cells^14^. This contrasts with B cells, where Ampk activation after 24 hours of stimulation persists during proliferation (Fig. 1A). In T cells, Ampk activation by nutrient limitation, metformin treatment, or AICAR, results in decreased *IFN* gene transcription and reduced T cell effector function^29^. Here, activation of Ampk by phenformin prevents GC differentiation and CSR, but still allows for generation of high-specificity antibodies (Fig. 6A–E).

To study Ampk during B cell activation *in vivo*, we made a B cell specific KO of the catalytic Ampk alpha subunit, *Prkaa1* that achieved >80% deletion efficiency, but did not detect a phenotype similar to B cell specific *Lkb1* KO mice^4^. In fact, Lkb1 loss increases CSR and decreases plasmablast differentiation, whereas Ampk loss instead decreases CSR and slightly increases plasmablast differentiation (Figs 2D, 4D). These results suggest that Lkb1 acts through substrates other than Ampk to regulate B cell activation and GC formation, or that other related Lkb1 targets may compensate for the loss of Ampk. For example, Lkb1 phosphorylates 13 other Ampk family member proteins including Mark, Brsk, and Nuak proteins^7^. Sik2 and Mark2 are Lkb1 targets that phosphorylate Crtc2^41^, a transcriptional co-activator of CREB required for GC exit^42^, and their redundant activity might compensate for the loss of Ampk to rescue plasma cell differentiation.

We were further surprised to find no evidence for Ampk in regulating metabolism in B cells. Nutrient uptake and routing was identical in WT and Ampk KO B cells (Figs 2G, 3). One possibility is that Ampk is dispensable for homeostatic nutrient handling but required for metabolic adaptations under stressful conditions not examined here. Supporting this idea, studies in T cells show that Ampk can regulate glutamine metabolism during glucose deprivation^29^ and total body knockout of Ampk makes B and T cells unable to survive ATP synthase inhibition with oligomycin^21^. Recent studies show that GCs in mice are hypoxic microenvironments^43^ and Ampk links to inflammation in hypoxia^44^. Resting and activated Ampk KO B cells express genes and gene profile signatures similar to WT B cells, except for two repressed transcripts, *Zfp318* and *Ighd*. This specificity is remarkable and replicates the specificity of *Zfp318* KO B cells, in which there were only two differentially expressed genes, *Ighd* and *Sva*, an antigen related to the *Vav-Cre* recombinase deletion construct^35^. Loss of *Ighd* transcripts parallels a loss of surface IgD protein expression in the Ampk KO B cells after stimulation. A possible but less likely contributor to a difference in later time point IgD expression between WT and Ampk KO B cells could be differential increase in cell death in the *in* vitro culture system between days 4 and 5 of stimulation. The role(s) of IgD in B cells remains elusive, as IgD is present at very low levels in human and rodent serum^45^, but has recently been suggested to modulate Th2 responses to soluble antigens through interactions with basophils^46^. Because of co-expression of IgM and IgD in immature B cells, IgD may sequester signaling molecules from IgM to inhibit BCR signaling^47^, and IgD may play a similar role in mature B cells. Regardless of the role of IgD, the specific regulation of *Zfp318* and *Ighd* by Ampk provides new insight into immunoglobulin gene regulation as a non-canonical role for Ampk.

## Materials and Methods

### Mice

C57BL/6J, *Prkaa1*^*fl/fl*^, *CD19-Cre*, and *Rosa26 lox-STOP-lox EYFP* mice (JAX: 000664, 014141, 006785, and 006148) were housed in a specific pathogen-free animal facility at UCLA. All studies were on mixed-sex mice between 6 to 16 weeks of age with approval from the UCLA Institutional Animal Research Committee (#1998-113-63C). All experiments were performed according to the National Institutes of Health and ARRIVE guidelines on the use of laboratory animals. Figures 1, 6 used WT C57BL/6J mice, and Figs 2–5 used B cell specific Ampk KO or Ampk WT littermate mice. Genotypes for WT (*Prkaa1*^+/+^) and Ampk KO (*Prkaa1*^−/−^) mice were as follows: *Prkaa1*^*wt/wt*^ or *Prkaa1*^*fl/fl*^, respectively, with *CD19-Cre*^+*/−*^, and *Rosa26-YFP*^+*/*+^ or ^+*/−*^ for experiments requiring YFP (Fig. 3B–D,F) or *Rosa26-YFP*^−/−^ for experiments without YFP tracer (Figs 3E,F, 4–6).

### Stimulation of isolated mouse B cells

Red blood cell-lysed mouse spleen cells were enriched for B cells using CD43 negative magnetic selection (Miltenyi). Cells were grown in RPMI1640 supplemented with 10% FBS and 50 μM β-mercaptoethanol. B cells were stimulated with 1 μg/ml anti-CD40 mAb (BD Pharmingen) and 25 ng/ml IL-4 (R&D Systems). At day 3, anti-CD40 was washed out and cells replated in medium containing only IL-4 until day 5. For early Ampk activation, phenformin (Sigma) was resuspended in 1x PBS, pH 7.4, and used at 100 µM, and A-769662 (Abbott) was resuspended in DMSO and used at 50 µM at the time of stimulation.

### Immunoblotting

Cells were lysed in Lysis Buffer containing 50 mM Tris HCl pH 7.4, 100 mM NaCl, 1 mM EDTA and 1% Triton X-100 supplemented with Protease Inhibitor and Phosphatase Inhibitor Cocktails 2 and 3 (Sigma). Extracts were quantified and denatured by boiling with DTT and 10–30 µg protein separated by SDS-PAGE and transferred to nitrocellulose before blocking in 5% milk in TBST. Membranes were incubated overnight in the indicated antibodies in 5% BSA in TBST. Membranes were then incubated in fluorescent secondary antibody and imaged using the Odyssey Fc imaging system (LI-COR). Complete antibody information can be found in the Supplemental Methods.

### Flow cytometry

Single cell suspensions were incubated with Fc Block (BD Pharmingen) at 1:500 for 15 min, then washed and stained for 20 min in 50 µl FACS buffer (2% FBS in PBS) on ice in the dark. Data were obtained on a BD LSRII or BD Fortessa (BD Biosciences) and analyzed with FlowJo software (Treestar). Antibodies were used at a 1:200 dilution. Complete antibody information can be found in the Supplemental Methods. Assessments of glucose import used a 2-NBDG Glucose Uptake Assay Kit (Biovision) according to the manufacturer’s instructions.

### Extracellular metabolite analysis

Glutamine levels in full media and after 24 hours of stimulation were measured by plating 10^6^ cells/ml in 2 ml, centrifuging to remove cells and debris, and analyzing using a BioProfile Basic Analyzer (NOVA Biomedical).

### Live cell interferometry

Live cell interferometry (LCI) was used to measure biomass accumulation rate^25^. Cells were plated at 7.5 × 10^5^ cells/ml on Poly-L-Lysine (Sigma) coated μ-Slide 2-Well Ph+ glass bottom slides (Ibidi) and imaged every 15 min for 72 h in a custom-built chamber as previously described^48^. LCI was performed on a Zeiss Axio Observer A1 with stage-top incubation system (Zeiss) using using a 20 × 0.4 NA objective. LCI data were captured with a SID4Bio (Phasics) QWLSI camera^49^. MATLAB (Mathworks) was used to analyze LCI data. Quantitative phase microscopy data was processed using SID4Processing (Phasics) to generate phase-shift images compatible with MATLAB. A custom MATLAB script was used to track the mass of individual cells as previously described^50^.

### Immunization and ELISA

Mice were immunized with 50 µg NP-(28)-CGG (Biosearch Technologies) in ImJect Alum (Thermo Scientific) via intra-peritoneal injection. Blood was collected at 14 days and assayed by ELISA. Serum Ig concentrations were determined using anti-mouse Ig as a capture antibody and developed with isotype-specific goat anti-mouse antibodies conjugated to HRP (Southern Biotech). Antigen-specific ELISA was performed by coating a plate with NP-(8)- or NP-(20)-BSA and developed with an isotype specific goat anti-mouse antibody conjugated to HRP. For immunizations with phenformin, mice were given water containing either vehicle (5 mg/ml sucralose, Sigma) or phenformin (1.8 mg/ml, Sigma) with vehicle in amber bottles one day before immunization, and water was changed twice per week.

### Metabolomics

B cells were grown for 24 hours in media with glucose or glutamine free RPMI (Gibco) supplemented with 2 g/L [U-^13^C] glucose or 3 mM [U-^13^C] glutamine, respectively (Cambridge Isotope Laboratories) and non-dialyzed FBS. Metabolites were extracted with cold 80% methanol and measured using Ultra High Performance Liquid Chromatography Mass Spectrometry (UHPLC-MS), as previously described^24,51,52^. To extract intracellular metabolites, cells were pelleted by centrifugation (1000 RPM, 4 °C) and rinsed with cold 150 mM ammonium acetate (pH 7.3), pelleted again, followed by addition of 1 ml cold 80% MeOH in water. To the cell suspensions, 10 nmol D/L-norvaline were added and rigorously mixed followed by centrifugation (1.3 × 10^4^ rpm, 4 °C). The supernatant was transferred into a glass vial and pellet was further extracted with 200 µl cold 80% MeOH in water. After centrifugation, supernatant was combined, metabolites dried down under vacuum, and resuspended in 70% acetonitrile. For the mass spectrometry-based analysis of the sample, 5 μl were injected onto a Luna NH2 (150 mm × 2 mm, Phenomenex) column. The samples were analyzed with an UltiMate 3000RSLC (Thermo Scientific) coupled to a Q Exactive mass spectrometer (Thermo Scientific). The Q Exactive was run with polarity switching (+3.50 kV/−3.50 kV) in full scan mode with an m/z range of 65–975. Separation was achieved using A) 5 mM NH4AcO (pH 9.9) and B) ACN. The gradient started at 15% A) going to 90% A) over 18 min, followed by an isocratic step for 9 min and reversal to the initial 15% A) for 7 min. Metabolites and isotopomers thereof were quantified with TraceFinder 3.3 using accurate mass measurements (≤3 ppm) and retention times. For isotopologue distribution measurements, data was corrected for naturally occurring ^13^C as described in^53^. Fractional contributions were calculated using the formula $FC=\frac{{\sum }_{0}^{n}i\ast {m}_{i}}{n{\sum }_{0}^{n}{m}_{i}}$ as described^54^, where *m*_*i*_ denotes the intensity of the isotopologue, and *n* marks the number of carbons in a given metabolite. Data were normalized to cell counts. Metabolite relative amounts, isotopomer distribution values, MSVA scores, and DPI lists are included in a supplemental excel file (Table S1).

### RNA extraction

At least 10^7^ WT and Ampk KO B cells were grown in biological triplicates and RNA purified immediately after isolation, or 24 hours after anti-CD40 plus IL-4 stimulation using the RNeasy Mini Kit (Qiagen) and RNase-free DNase (Qiagen) following the manufacturer’s protocols. All samples showed an A260/280 ratio >1.99. Prior to library preparation, quality control of the RNA was performed using the Advanced Analytical Technologies Fragment Analyzer (Advanced Analytical, Inc.) and analyzed using PROSize 2.0.0.51 software. RNA Quality Numbers (RQNs) were computed per sample between 8.7 and 10, indicating intact total RNA per sample prior to library preparation.

### RNA-seq library preparation

Strand-specific ribosomal RNA (rRNA) depleted RNA-Seq libraries were prepared from 1 µg of total RNA using the KAPA Stranded RNA-Seq Kit with Ribo-Erase (Kapa Biosystems, Roche). Briefly, rRNA was depleted from total RNA samples, the remaining RNA was heat fragmented, and strand-specific cDNA was synthesized using a first strand random priming and second strand dUTP incorporation approach. Fragments were then A-tailed, adapters were ligated, and libraries were amplified using high-fidelity PCR. All libraries were prepared in technical duplicates per sample and resulting raw sequencing reads merged for downstream alignment and analysis. Libraries were paired-end sequenced at 2 × 150 bp on an Illumina NovaSeq 6000.

Lists of transcript/gene-level expression values, KO signature ORA results, and differentiation signature GSVA results are included in a supplemental excel file (Table S2).

### Statistical analyses

All metabolomics and transcriptomics statistical analyses are described in the above methods. Values represent mean ± S.D. or S.E.M. Data were analyzed with Prism 6 (GraphPad) (Figs 1–3, 6), MATLAB (Mathworks) (Fig. 1E–G) or R (Figs 3–5). Parametric data were analyzed using unpaired two-tailed Student’s t-tests, or 2-way ANOVA with Bonferroni correction for multiple comparisons. Transcriptomic volcano and kinetic time course expression plots were analyzed using DESeq2 Wald tests with Benjamini-Hochberg FDR correction for multiple comparisons. For all data sets, *P* ≤ 0.05 was considered significant. *P ≤ 0.05, **P ≤ 0.01, ***P ≤ 0.001, ****P ≤ 0.0001.

## Supplementary information

Supplementary Materials

Table S1

Table S2

## Data Availability

All raw RNA-Seq reads, transcript abundance values, and processed gene count matrices were submitted to the NCBI Gene Expression Omnibus (GEO) under accession GSE121025. All metabolomics and downstream transcriptomics datasets have been provided as supplemental material in this study (Tables S1 and S2).

## Code Availability

All custom code used for metabolomic/transcriptomic analyses are available on Atlassian BitBucket at http://bitbucket.org/ahsanfasih/AmpkBcell/src/master and detailed descriptions of data analysis can be found in the Supplementary Methods.
