# Supplementary material for: Ampk regulates IgD expression but not energy stress with B cell activation
Source: Sci Rep. 2019 Jun 3;9:8176. doi: 10.1038/s41598-019-43985-y (PMC6546716; doi:10.1038/s41598-019-43985-y)
Supplement: Supplementary file 1 — Supplementary Materials [file 41598_2019_43985_MOESM1_ESM.docx]

**Ampk regulates IgD expression but not energy stress with B cell activation**

Lynnea R. Waters, Fasih M. Ahsan, Johanna ten Hoeve, Jason S. Hong, Diane N.H. Kim, Aspram Minasyan, Daniel Braas, Thomas G. Graeber, Thomas A. Zangle, and Michael A. Teitell

**SUPPLEMENTARY INFORMATION**

**Supplementary Figures**

- **Supplementary Figure 1.** Full-length blots/gels for Figure 1A.
- **Supplementary Figure 2.** Full-length blots/gels for Figure 2C.

**Supplementary Methods**

- Antibody table. Related to Figures 1, 2, and 6.
- Data analysis for metabolomics and RNA sequencing. Related to Figures 3, 4, and 5.

**Supplementary Data Files**

- **Table S1.** List of relative metabolite amounts, and isotopomer distribution results from *Prkaa1^-/-^* and WT naïve and stimulated B cells with ^13^C_6_-glucose or ^13^C_5_-glutamine labeling. Related to Figure 3.
- **Table S2.** List of transcript/gene-level expression values, GO term overrepresentation results, and signature differentiation pathway scores from RNA-Seq datasets for WT and Ampk KO B cells over 5 days of differentiation under GEO: GSE121025. Related to Figure 4 and 5.

**Supplementary References**

**SUPPLEMENTARY FIGURES**

Phospho-Ampkα (T172). CST 40H9^1^

0 / 6 / 12 / 18 / 24 / 48 / 72


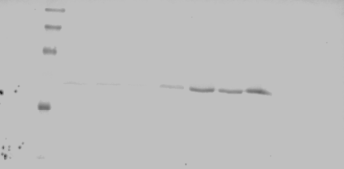


90 kDa

50 kDa

50 kDa

90 kDa

50 kDa

Total Ampkα. SCBT C-20^2^.


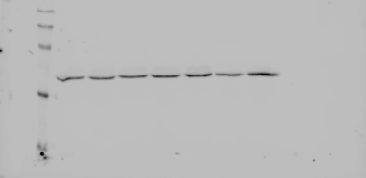


0 / 6 / 12 / 18 / 24 / 48 / 72

β-Tubulin. Sigma T4026^3^.


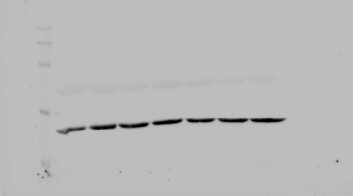


0 / 6 / 12 / 18 / 24 / 48 / 72

**Supplementary Figure 1.** Related to Figure 1A. Lane 1: Chameleon™ Duo Ladder (LICOR), Lane 2: WCL from naïve B cells, Lane 3: WCL from 6 hr anti-CD40/IL-4 stimulated B cells, Lane 4: WCL from 12 hr anti-CD40/IL-4 stimulated B cells, Lane 5: WCL from 18 hr anti-CD40/IL-4 stimulated B cells, Lane 6: WCL from 24 hr anti-CD40/IL-4 stimulated B cells, Lane 7: WCL from 48 hr anti-CD40/IL-4 stimulated B cells, Lane 8: WCL from 72 hr anti-CD40/IL-4 stimulated B cells.

Phospho-Ampkα (T172). CST 40H9^1^


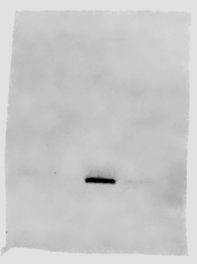


70 kDa

50 kDa

WT Ampk KO

Day 0 1 0 1

Total Ampkα. SCBT C-20^2^. (Two exposures)


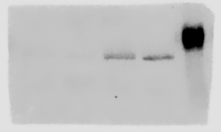

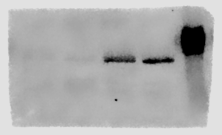


WT Ampk KO

Day 0 1 0 1

Day 0 1 0 1

WT Ampk KO

70 kDa

β-Tubulin. Sigma T4026^3^.


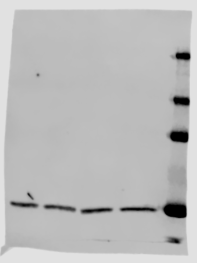


WT Ampk KO

Day 0 1 0 1

50 kDa

**Supplementary Figure 2.** Related to Figure 2C. Lane 1: Chameleon™ Duo Ladder (LICOR). Lane 2: WCL from naïve WT B cells. Lane 3: WCL from 24 hour stimulated WT B Cells. Lane 4: WCL from naïve Ampk KO B cells. Lane 5: WCL from 24 hour stimulated Ampk KO B cells. Note that membrane was cut after probing for P-Ampk T172 and β-Tubulin before stripping and reprobing for total Ampk.

**SUPPLEMENTARY METHODS**

**Antibodies**

| **Vendor** | **Antibody** | **Clone** | **Catalog** | **Use** |
| --- | --- | --- | --- | --- |
| BD Pharmingen | PE Rat Anti-Mouse CD45R/B220 | RA3-6B2 | 553090 | Flow cytometry |
| BD Pharmingen | PE Hamster Anti-Mouse CD95/Fas | Jo2 | 554258 | Flow cytometry |
| BD Pharmingen | PE Rat Anti-Mouse CD138 (Syndecan-1) | 281-2 | 553714 | Flow cytometry |
| Invitrogen | CD45R (B220) Monoclonal Antibody (RA3-6B2), Alexa Fluor 700, eBioscience™ | RA3-6B2 | 56-0452-82 | Flow cytometry |
| BD Horizon | V450 Rat Anti-Mouse CD86 | GL1 | 560450 | Flow cytometry |
| BD Horizon | V450 Hamster Anti-Mouse CD69 | H1.2F3 | 560690 | Flow cytometry |
| BD Horizon | V450 Rat Anti-Mouse IgG1 | A85-1 | 562107 | Flow cytometry |
| Invitrogen | MHC Class II (I-A/I-E) Monoclonal Antibody (M5/114.15.2), Alexa Fluor 700, eBioscience™ | M5/114.15.2 | 56-5321-82 | Flow cytometry |
| Invitrogen | IgD Monoclonal Antibody (11-26c (11-26)), eFluor 450, eBioscience™ | 11-26c(11-26) | 48-5993-82 | Flow cytometry |
| Invitrogen | GL7 Monoclonal Antibody (GL-7 (GL7)), eFluor 450, eBioscience™ | GL-7 (GL7) | 48-5902-82 | Flow cytometry |
| BD Pharmingen | Purified NA/LE Rat Anti-Mouse CD16/CD32 (Fc Block) | 2.4G2 | 553140 | Flow cytometry |
| BD Pharmingen | Purified NA/LE Hamster Anti-Mouse CD40 | HM40-3 | 553721 | Stimulation |
| Cell Signaling Technologies | Phospho-AMPKα (Thr172) (40H9) Rabbit mAb; 1:1000 | 40H9 | 2535S | Western blot |
| Santa Cruz Biotechnology | AMPKα1 Goat Polyclonal Antibody (C-20); 1:100 | C-20 | Sc-19128 | Western blot |
| Millipore-Sigma | Monoclonal Anti-β-Tubulin antibody produced in mouse ascites fluid; 1:1000 | TUB 2.1 | T4026 | Western blot |

**Metabolomics data analysis.** Data analysis, including principal components analysis (PCA) and clustering, was performed using the statistical language R v3.4.4 and Bioconductor v3.6.0 packages. Metabolite abundance was normalized per µg of protein content per metabolite extraction, and metabolites not detected were set to zero. Metabolite normalized amounts were scaled and centered into Z-scores for relative comparison using R base function scale() with parameters “scale = TRUE, center = TRUE”. Volcano plots were prepared using R package ggplot2 v. 2.2.1; adjusted *P* values were calculated using R base functions t.test() and p.adjust() with parameter “method = ‘BH’” using corrected isotopomer distribution (MID) values.

PCA was performed using R packages FactoMineR v1.34 and factoextra v1.0.5. Normalized metabolite amounts were standardized using a log_2_(normalized amounts + 1) transformation, and PC scores computed with function PCA() using parameters “scale.unit = TRUE, ncp = 10, graph = FALSE”. PCA individual score plots were displayed using function fviz_pca(). PCA variable loadings plots were generated using function fviz_pca_var(), extracting metabolite scores for contributions to the top ten principal components, strength of representation on the factor map (cosine2) and variable coordinates indicating Pearson correlation coefficient *r* of each metabolite to the top ten principal components. For whole relative amounts PCA, variable loadings were classified into k=4 separate clusters using k-means clustering in function kmeans() using parameters “set.seed(123), centers = 4 , nstart 25”. K-means clustering of glutamine and glucose MID tracing variable loadings were perform using k=2 clusters with parameters “set.seed(123), centers = 2, nstart = 25”.

Pathway-level relative amounts metabolite set variation analysis (MSVA) was performed using R Bioconductor package GSVA v1.26.0^7^. Metabolite normalized relative abundances were standardized using a log_2_(normalized amounts + 1) transformation, and metabolites per sample were converted to a pathways per sample matrix using function gsva() with parameters “method = gsva, rnaseq = FALSE, abs.ranking = FALSE, min.sz = 5, max.sz = 500”. GSVA pathway enrichment scores were then extracted and significance testing between conditions was calculated using R Bioconductor package limma v3.34.9, fitting a linear model to each metabolite and assessing differences in normalized abundance using an empirical Bayes moderated F-statistic with an adjusted *P* value threshold of 0.05, using the Benjamini-Hochberg false discovery rate of 0.05^8,9^. Pathway metabolite sets were constructed using the KEGG Compound Database and derived from the existing Metabolite Pathway Enrichment Analysis (MPEA) toolbox ^10,11^. Assessment of differentially produced isotopomers (DPI) was calculated between time point (D1/D0, all samples) or genotype (KO/WT, all samples), using R base function t.test, and corrected for multiple testing using p.adjust() with parameter “method = ‘BH’”. DPIs were threshold at *P* < 0.05 or adjusted *P* < 0.05.

Metabolite relative amounts, isotopomer distribution values, MSVA scores, and DPI lists are included in a supplemental excel file (Table S1).

**RNA-Seq data processing.** Raw sequencing runs were filtered for low quality reads and adapter contamination using FastQC (<http://www.bioinformatics.babraham.ac.uk/projects/fastqc>), SeqTK (https://github.com/lh3/seqtk), and Cutadapt^12^. rRNA contaminants carried over from the rRNA-depletion library preparation were filtered and removed using BBDuk from the BBTools (v. 38.08) suite (https://jgi.doe.gov/data-and-tools/bbtools/), comparing reads to rRNA species located in the SILVA rRNA database^13^. Filtered reads were quantified and quasi-mapped to the *Mus musculus* Gencode M17 (GRCm38.p6) reference transcriptome using the alignment-free transcript level quantifier Salmon v.0.9.1^14-16^. The resulting estimated transcript counts were summarized into normalized gene level transcripts per million (TPM) and estimated count matrices using R (v. 3.4.0) Bioconductor (v. 3.5) package tximport (v. 1.4)^17^.

The resulting sample gene count matrix was normalized and analyzed for differential gene expression using R (v. 3.4.0) Bioconductor (v. 3.5) package DESeq2 v1.16.0^18,19^. Significance testing was performed using the Wald test, testing for the significance of deviance in a full design “Batch + Genotype-Stimulation”, modeling the genotype effect at each stimulated and unstimulated timepoint while accounting for batch variance between matched stimulation replicates. Resulting *P* values were adjusted for multiple testing using the Benjamini-Hochberg procedure^8^. DEGs were filtered using an adjusted false discovery rate (FDR) *P* value < 0.05 and an absolute log_2_(Fold Change) > 0.5 in either the stimulated or unstimulated paired conditions. KO signatures were prepared by intersecting the list of all DEGs at each timepoint between day 1 to day 5 stimulation. GO term over-representation analysis (ORA) was performed for the KO signature using R package clusterProfiler v. 3.6.0 using default parameters and the organism database org.Mm.eg.db v. 3.5.0 in function enrichGO()^20^.

Volcano plots were made with ggplot2 as described above, using the DESeq2 output adjusted *P* values and log_2_-fold changes per KO/WT comparison at each timepoint. Time course kinetic plots were made using ggplot2 with DESeq2 size factor normalized counts using function plotCounts(). Gene expression heat maps were prepared using pheatmap() with row Z-scores calculated as the variance stabilized transform (VST) subtracted by the row mean. PCA was plotted using VST values with function plotPCA() with parameter “ntop = 100000”. GSVA on B cell differentiation signatures were performed similarly to metabolomics data as noted above. Pathway-level signature gene set enrichment analysis was performed using R Bioconductor package GSVA v1.26.0 function *gsva()* with parameters *“method = gsva, rnaseq = FALSE, abs.ranking = FALSE, min.sz = 5, max.sz = 500”* using a log_2_(TPM + 1) transformed gene expression matrix^7^. GSVA pathway enrichment scores per sample were extracted and assessed for significance using R Bioconductor package limma v3.34.9, as described above except with a Benjamini-Hochberg adjusted *P* value threshold = 0.01. Differentiation signature gene sets was obtained for 24 h anti-CD40 plus IL4 activated B cells^21,22^, GC B cells^22,23^, and ASCs^22,23^. The top 55 genes for each signature were selected by adjusted *P* value ranks between WT day 0 - WT day 2 (activation), WT day 0 - WT day 3 (GC), and WT day 0 – WT day 5 (ASC).

Lists of transcript/gene-level expression values, KO signature ORA results, and differentiation signature GSVA results are included in a supplemental excel file (Table S2).

**SUPPLEMENTARY DATA FILES**

- **Table S1.** List of relative metabolite amounts, and isotopomer distribution results from *Prkaa1^-/-^* and WT naïve and stimulated B cells with ^13^C_6_-glucose or ^13^C_5_-glutamine labeling. Related to Figure 3.
- **Table S2.** List of transcript/gene-level expression values, GO term overrepresentation results, and signature differentiation pathway scores from RNA-Seq datasets for WT and Ampk KO B cells over 5 days of differentiation under GEO: GSE121025. Related to Figure 4 and 5.

**SUPPLEMENTARY REFERENCES**

1 Zhang, C. S. *et al.* Fructose-1,6-bisphosphate and aldolase mediate glucose sensing by AMPK. *Nature* **548**, 112-116, doi:10.1038/nature23275 (2017).

2 Ben Sahra, I. *et al.* The antidiabetic drug metformin exerts an antitumoral effect in vitro and in vivo through a decrease of cyclin D1 level. *Oncogene* **27**, 3576-3586, doi:10.1038/sj.onc.1211024 (2008).

3 Yang, C. Y. *et al.* Dual-specificity phosphatase 14 (DUSP14/MKP6) negatively regulates TCR signaling by inhibiting TAB1 activation. *J Immunol* **192**, 1547-1557, doi:10.4049/jimmunol.1300989 (2014).

4 MacLean, B. *et al.* Skyline: an open source document editor for creating and analyzing targeted proteomics experiments. *Bioinformatics* **26**, 966-968, doi:10.1093/bioinformatics/btq054 (2010).

5 Crooks, G. E., Hon, G., Chandonia, J.-M. & Brenner, S. E. WebLogo: A Sequence Logo Generator. *Genome Research* **14**, 1188-1190 (2004).

6 Herzig, S. & Shaw, R. J. AMPK: guardian of metabolism and mitochondrial homeostasis. *Nat Rev Mol Cell Biol* **19**, 121-135, doi:10.1038/nrm.2017.95 (2018).

7 Hanzelmann, S., Castelo, R. & Guinney, J. GSVA: gene set variation analysis for microarray and RNA-seq data. *BMC Bioinformatics* **14**, 7, doi:10.1186/1471-2105-14-7 (2013).

8 Benjamini, Y. & Hochberg, Y. Controlling the False Discovery Rate - a Practical and Powerful Approach to Multiple Testing. *J Roy Stat Soc B Met* **57**, 289-300 (1995).

9 Ritchie, M. E. *et al.* limma powers differential expression analyses for RNA-sequencing and microarray studies. *Nucleic Acids Res* **43**, e47, doi:10.1093/nar/gkv007 (2015).

10 Kanehisa, M., Goto, S., Sato, Y., Furumichi, M. & Tanabe, M. KEGG for integration and interpretation of large-scale molecular data sets. *Nucleic Acids Res* **40**, D109-114, doi:10.1093/nar/gkr988 (2012).

11 Kankainen, M., Gopalacharyulu, P., Holm, L. & Oresic, M. MPEA--metabolite pathway enrichment analysis. *Bioinformatics* **27**, 1878-1879, doi:10.1093/bioinformatics/btr278 (2011).

12 Martin, M. Cutadapt removes adapter sequences from high-throughput sequencing reads. *2011* **17**, doi:10.14806/ej.17.1.200 pp. 10-12 (2011).

13 Quast, C. *et al.* The SILVA ribosomal RNA gene database project: improved data processing and web-based tools. *Nucleic Acids Res* **41**, D590-596, doi:10.1093/nar/gks1219 (2013).

14 Harrow, J. *et al.* GENCODE: the reference human genome annotation for The ENCODE Project. *Genome Res* **22**, 1760-1774, doi:10.1101/gr.135350.111 (2012).

15 Mudge, J. M. & Harrow, J. Creating reference gene annotation for the mouse C57BL6/J genome assembly. *Mamm Genome* **26**, 366-378, doi:10.1007/s00335-015-9583-x (2015).

16 Patro, R., Duggal, G., Love, M. I., Irizarry, R. A. & Kingsford, C. Salmon provides fast and bias-aware quantification of transcript expression. *Nat Methods* **14**, 417-419, doi:10.1038/nmeth.4197 (2017).

17 Soneson, C., Love, M. I. & Robinson, M. D. Differential analyses for RNA-seq: transcript-level estimates improve gene-level inferences. *F1000Res* **4**, 1521, doi:10.12688/f1000research.7563.2 (2015).

18 Love, M. I., Huber, W. & Anders, S. Moderated estimation of fold change and dispersion for RNA-seq data with DESeq2. *Genome Biol* **15**, 550, doi:10.1186/s13059-014-0550-8 (2014).

19 Huber, W. *et al.* Orchestrating high-throughput genomic analysis with Bioconductor. *Nat Methods* **12**, 115-121, doi:10.1038/nmeth.3252 (2015).

20 Yu, G., Wang, L. G., Han, Y. & He, Q. Y. clusterProfiler: an R package for comparing biological themes among gene clusters. *OMICS* **16**, 284-287, doi:10.1089/omi.2011.0118 (2012).

21 Wu, Y. L., Stubbington, M. J., Daly, M., Teichmann, S. A. & Rada, C. Intrinsic transcriptional heterogeneity in B cells controls early class switching to IgE. *J Exp Med* **214**, 183-196, doi:10.1084/jem.20161056 (2017).

22 Shi, W. *et al.* Transcriptional profiling of mouse B cell terminal differentiation defines a signature for antibody-secreting plasma cells. *Nat Immunol* **16**, 663-673, doi:10.1038/ni.3154 (2015).

23 Gloury, R. *et al.* Dynamic changes in Id3 and E-protein activity orchestrate germinal center and plasma cell development. *J Exp Med* **213**, 1095-1111, doi:10.1084/jem.20152003 (2016).
